# Synthesis of Conjugated Polymers Containing B←N Bonds with Strong Electron Affinity and Extended Absorption

**DOI:** 10.3390/polym11101630

**Published:** 2019-10-09

**Authors:** Bo Pang, Zhonghai Tang, Yongchun Li, Huifeng Meng, Ying Xiang, Yuqing Li, Jianhua Huang

**Affiliations:** 1 College of Materials Science and Engineering, Huaqiao University, Xiamen 361021, China; 17013081023@hqu.edu.cn (B.P.); xliyongchuny@163.com (Y.L.); huifengsuc@163.com (H.M.); 18013081036@hqu.edu.cn (Y.X.); 17013081014@hqu.edu.cn (Y.L.); 2 Beijing National Laboratory for Molecular Sciences, Institute of Chemistry, Chinese Academy of Sciences, Beijing 100190, China; tzhzhonghai@163.com

**Keywords:** B←N bond, conjugated polymer, depressed energy level, electronic device

## Abstract

The B←N is isoelectronic to the C–C, with the former having stronger dipole moment and higher electron affinity. Replacing the C–C bonds in conjugated polymers with B←N bonds is an effective pathway toward novel polymers with strong electron affinity and adjustable optoelectronic properties. In this work, we synthesize a conjugated copolymer, namely, BNIDT-DPP, based on a B←N embedded unit, BNIDT, and a typical electron-deficient unit, diketopyrrolopyrrole (DPP). For comparison, the C–C counterpart, i.e., IDT-DPP, is also synthesized. In contrast to IDT-DPP, the B←N embedded polymer BNIDT-DPP shows an extended absorption edge (836 versus 978 nm), narrowed optical bandgap (1.48 versus 1.27 eV), and higher electron affinity (3.54 versus 3.74 eV). The Gaussian simulations reveal that the B←N embedded polymer BNIDT-DPP is more electron-deficient in contrast to IDT-DPP, supporting the decreased bandgap and energy levels of BNIDT-DPP. Organic thin-film transistor (OTFT) tests indicate a well-defined p-type characteristic for both IDT-DPP and BNIDT-DPP. The hole mobilities of IDT-DPP and BNIDT-DPP tested by OTFTs are 0.059 and 0.035 cm^2^/V·s, respectively. The preliminary fabrication of all-polymer solar cells based on BNIDT-DPP and PBDB-T affords a PCE of 0.12%. This work develops a novel B←N embedded polymer with strong electron affinity and extended absorption, which is potentially useful for electronic device application.

## 1. Introduction

Conjugated polymers have been intensively studied for application to organic electronics, e.g., organic field effect transistors (OFETs) and organic solar cells (OSCs) due to their light weight, flexibility, low cost, and easy processability [1,2,3,4,5,6]. The performance of these devices is fundamentally determined by the intrinsic photophysics and frontier molecular orbital (FMO) energy levels of conjugated polymers. Adjusting these optoelectronic properties to a desirable level for specific device applications can be realized feasibly through structural tailoring. Donor–Acceptor (D-A) alternating copolymerization is the mainstream strategy to construct conjugated polymers, tailoring the D or A units to be straight and effective to adjust the optoelectronic properties [7]. Particularly, introducing heteroatoms, e.g., O, S, N, B, and P into the π-conjugated backbone can produce various polycyclic heteroaromatic units with pretty co-planarity and customizable optical properties and energy levels, which are promising D or A units that are potentially useful for building the conjugated polymers [8]. Among these heteroaromatic units, the B←N embedded electronic moieties have emerged as a brilliant class of functional molecules possessing unique properties [9,10,11,12,13,14,15,16,17,18,19,20,21,22,23]. As the B←N coordination bond is isoelectronic to the C–C covalent bond, introducing B←N bonds into the conjugated backbone is highlighted by several advantages. For example, the replacing a C–C by a B←N bond would maintain the π-electronic number of the backbone and obtain a conjugated framework with good co-planarity. Moreover, due to the strong electron-withdrawing property of B, the conjugated units containing B←N bonds usually have strong electron affinity with depressed energy levels in contrast to their C–C counterparts [10]. Additionally, in comparison to the non-polar C–C bond, the B←N is strongly polar, which would presumably lead to enhanced dipole–dipole interactions between the conjugated backbones. To this end, most B←N embedded molecules are potential A units to build the conjugated polymers.

**Scheme 1:**
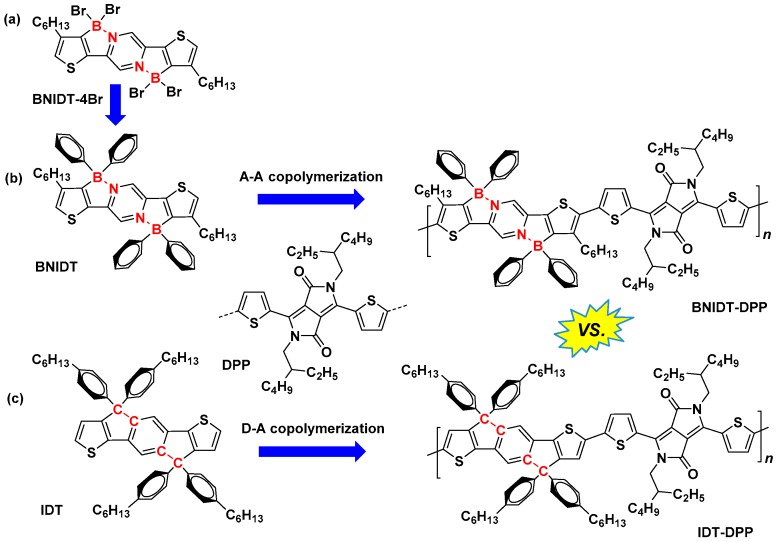
Molecular structures of IDT, BNIDT, DPP, IDT-DPP, and BNIDT-DPP. BNIDT: (**a**) B←N embedded unit, DPP: diketopyrrolopyrrole, IDT: indacenodithiophene.

Indacenodithiophene (IDT) is a typical ladder-type unit that has been widely used to construct conjugated polymers for OFETs and OSCs applications [24,25,26,27,28,29,30,31,32,33,34,35,36,37,38,39,40,41]. Recently, we embedded the B←N bonds into the IDT, disclosing a series of BNIDT units with low-lying energy levels, extended absorption, and decreased optical bandgaps (Scheme 1a) [18]. Unfortunately, these B←N embedded units are fragile to the moisture due to the strong electron-deficient property of four coordination B originated from the Br substitutions, and cannot be used to copolymerize with other D or A co-units. In this work, by replacing the Br atoms with phenyl (Ph) groups on the B, a chemically stable BNIDT unit was obtained (Scheme 1b). Then, this B←N embedded unit was copolymerized with another classic A unit, diketopyrrolopyrrole (DPP), to obtain an A–A copolymer, namely, BNIDT-DPP. DPP was selected as the co-monomer owing to its rigid backbone, good co-planarity, strong electron deficiency, and wide absorption spectra, which has been widely used for OFETs and OSC materials [42,43]. Moreover, a copolymer based on IDT and DPP, namely, IDT-DPP, was also synthesized for comparison to study the influence of B←N on the optoelectronic properties of the conjugated polymers (Scheme 1c). It′s noticeable that the side groups of IDT-DPP and BNIDT-DPP are slightly different, with the hexyl chains anchoring on the Ph groups and β-positions for IDT and BNIDT, respectively. As the backbone modification using B←N bonds to replace the C–C bonds can impose strong influence on the optoelectronic properties, the effects of slightly different side chains on the absorption and energy levels can be ignored [18]. For the molecular conformations, packing order, and mobilities, the influence of side chains will be taken into account. By introducing B←N bonds into the polymer backbone, the energy levels were depressed, and the absorption profiles were extended greatly for BNIDT-DPP in contrast to the IDT-DPP. Furthermore, the OFETs and OSCs devices were prepared using these polymers. 

## 2. Results and Discussion 

**Scheme 2:**
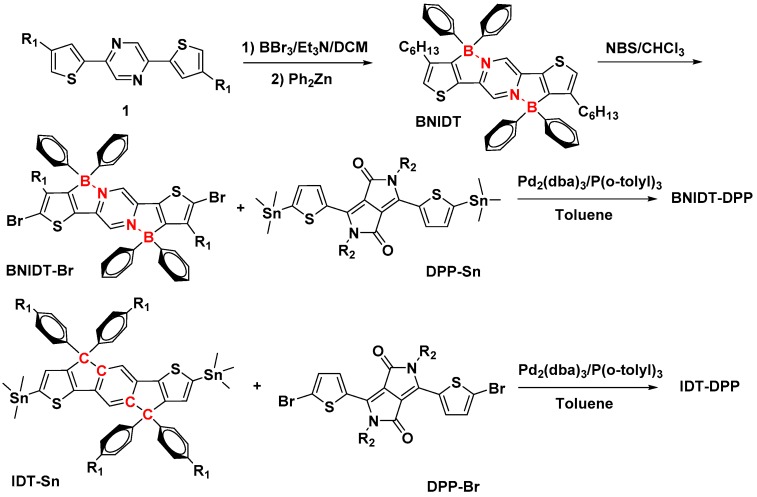
Synthetic routes toward the IDT-DPP and BNIDT-DPP.

**Figure 1:**
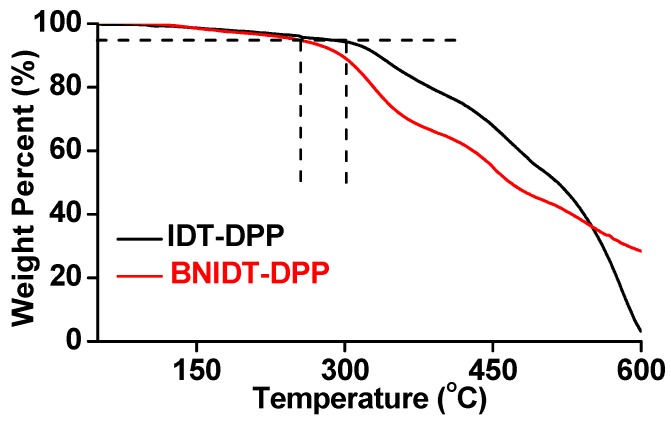
Thermogravimetric analysis (TGA) curves of IDT-DPP and BNIDT-DPP.

The synthetic routes of the two polymers are shown in Scheme 2. The starting compound 1 was subjected to BBr_3_ and Et_3_N in the dichloromethane (DCM) solution for the electrophilic borylation, and then Ph_2_Zn was added to produce the Ph-substituted BNIDT unit. The *N*-bromosuccinimide (NBS) was used to brominate the BNIDT at the α-position of thiophene, leading to BNIDT-Br. Finally, Pd-catalyzed Stille coupling polymerization was applied to alternately connect the BNIDT-Br and DPP-Sn, obtaining the A–A polymer BNIDT-DPP. For comparison, the D–A copolymer of IDT-DPP was also synthesized using two commercialized monomers of IDT-Sn and DPP-Br. These two polymers are readily soluble in commonly used solvents, e.g., DCM, chloroform (CF), tetrahydrofuran (THF), chlorobenzene (CB), and dichlorobenzene (DCB), indicating good solution processability. The number-average molecular weights (*M*_n_) tested by gel permeation chromatography (GPC, Waters, Milford City, USA) are 92.1 and 27.8 kDa (Table 1), respectively for IDT-DPP and BNDIT-DPP. Similar polydispersity indexes (PDI) of 2.09 and 2.31 are found for IDT-DPP and BNIDT-DPP, respectively. It’s worth to note that the *M*_n_ of IDT-DPP is threefold higher than that of BNIDT-DPP, presumably due to the weaker reactivity of the BNIDT in contrast to the IDT. To this end, the number-average degrees of polymerization (DP) estimated from *M*_n_/*M*_r_, where *M*_r_ is the molecular weight of one repeating unit, are 64 and 21, respectively, for IDT-DPP and BNIDT-DPP. As the DP of the polymer is one of the important factors to affect the device performance, the significant diversity of DP values between these two molecules would presumably influence their performance. The thermal stability was tested by thermogravimetric analysis (TGA, shimadzu corp., Kyoto, Japan) under N_2_ atmosphere (Figure 1). The 5% weight-loss temperature was tested to be 294 and 253 °C, respectively, for IDT-DPP and BNIDT-DPP, suggesting the good thermal stability of these polymers.

Before characterizing the optoelectronic properties of polymers, we compared the absorption profiles and energy levels of BNIDT-4Br and BNIDT to get insight into the effects of B-substitutions on the bandgaps and electron affinity [18,44]. As shown in Figure S1a (in Supplementary Materials), in contrast to the BNIDT-4Br, the Ph-substituted BNIDT show blue-shifted absorption in both solutions and films. The optical bandgap (*E*_g_^opt^) of BNIDT (1.95 eV) is also enlarged in comparison to the BNIDT-4Br (1.68 eV). Moreover, the FMO energy levels of BNIDT are also increased compared to these values of BNIDT-Br (Figure S1b in Supplementary Materials). In particular, the electron affinity of BNIDT is 3.79 eV, which is smaller than that of BNIDT-Br (4.71 eV) by 0.92 eV. These results indicate that by replacing the Br groups on the B positions with aromatic Ph groups, the absorption is blue-shifted, *E*_g_^opt^ is narrowed, and the electron affinity is reduced. This can be ascribed to the different electron-deficient properties of Br and Ph groups. Br is a strong electron-deficient group, whereas Ph is a conjugated group with electron-donor property. Accordingly, replacing the Br groups with Ph groups on the B positions could reduce the electron affinity and enhance the moisture tolerance of the backbone. Importantly, in contrast to the IDT unit, the Ph-substituted BNIDT has deeper energy levels and red-shifted absorption, which can be useful in constructing polymers with strong electron affinity and extended absorption.

**Figure 2:**
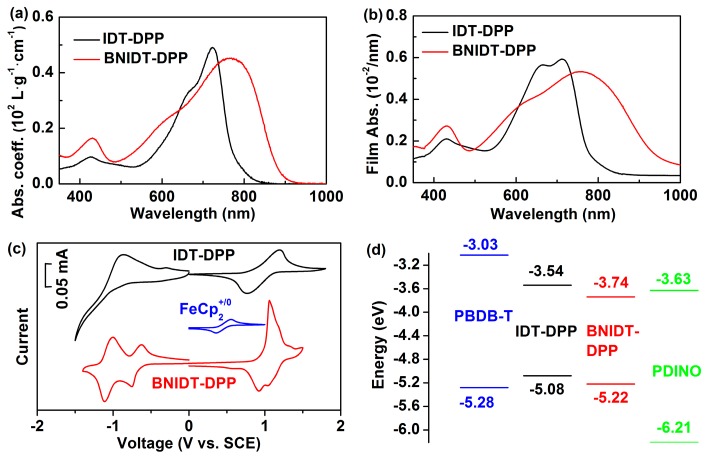
UV-vis-near infrared (NIR) absorption spectra in solutions (**a**) and films (**b**); CV curves (**c**); and energy level alignments (**d**) of PBDB-T, PDINO, IDT-DPP and BNIDT-DPP.

The UV-vis-NIR absorption spectra of IDT-DPP and BNIDT-DPP were tested, as shown in Figure 2 and summarized in Table 2. In diluted solutions, IDT-DPP shows an absorption peak (*λ*_max_^s^) at 720 nm, an absorption edge (*λ*_edge_^s^) of 825 nm, and a full width at half maximum (FWHM) of 110 nm (Figure 2a). Remarkably, after the B←N bonds were introduced into the backbone, the BNIDT-DPP displays a significantly red-shifted *λ*_max_^s^ of 784 nm, a *λ*_edge_^s^ of 929 nm, and an enhanced FWHM value of 277 nm. The identical trend was found in the film absorption from IDT-DPP to BNIDT-DPP (Figure 2b). The *E*_g_^opt^ estimated from the film absorption edge (*λ*_edge_^f^) of IDT-DPP (836 nm) and BNIDT-DPP (978 nm) is 1.48 and 1.27 eV, respectively. These results indicate that upon introducing B←N bonds into the polymer backbone, the absorption spectra apparently red-shift and extend to near infrared (ca. 1000 nm), and the bandgaps obviously decrease. From solutions to films, the FWHMs increase and *λ*_edge_ red-shift for both IDT-DPP and BNIDT-DPP, indicating the strong π–π interactions between polymer backbones in films. However, the absorption peaks (*λ*_max_) blue-shift from solutions to films for the two polymers, which was probably due to the H-aggregation type interchain stacking in the solid state [45].

Cyclic voltammetry (CV) was tested to study the electrochemical properties and estimate the FMO energy levels. As shown in Figure 2c, IDT-DPP demonstrates a reversible oxidation process, whereas the reduction process is irreversible. For BNIDT-DPP, both oxidation and reduction processes are reversible or quasi-reversible, indicating the high electrochemical stability of BNIDT-DPP. The FMO energy levels are evaluated by the formula *E*_HOMO/LUMO_ = −(4.8 − E_(Fc0/+)_ + *E*_ox/red_^onset^). As summarized in Table 2 and shown in Figure 2d, the highest occupied molecular orbital (HOMO) and lowest unoccupied molecular orbital (LUMO) of IDT-DPP are calculated to be –5.08 and –3.54 eV, respectively. After embedding B←N bonds into the backbone, BNIDT-DPP displays significantly lowered HOMO (–5.22 eV) and LUMO (–3.74 eV). Particularly, from IDT-DPP to BNIDT-DPP, the HOMOs decrease by 0.14 eV, whereas the LUMOs reduce by 0.20 eV, comprehensively leading to a depressed bandgap (*E*_g_^CV^) by 0.06 eV. The CV tests demonstrate that compared to IDT-DPP, the B←N embedded polymer BNIDT-DPP has decreased FMO energy levels, a narrowed bandgap, and stronger electrochemical stability. These results mainly originated from the electron-withdrawing property of B in the polymer backbone, enhancing the electron affinity and stability, as will be confirmed by the theoretical calculations. It’s worth noticing that the deep LUMO of BNIDT-DPP confirms its strong electron affinity, which is desirable for the electron acceptor of polymer solar cells.

**Figure 3:**
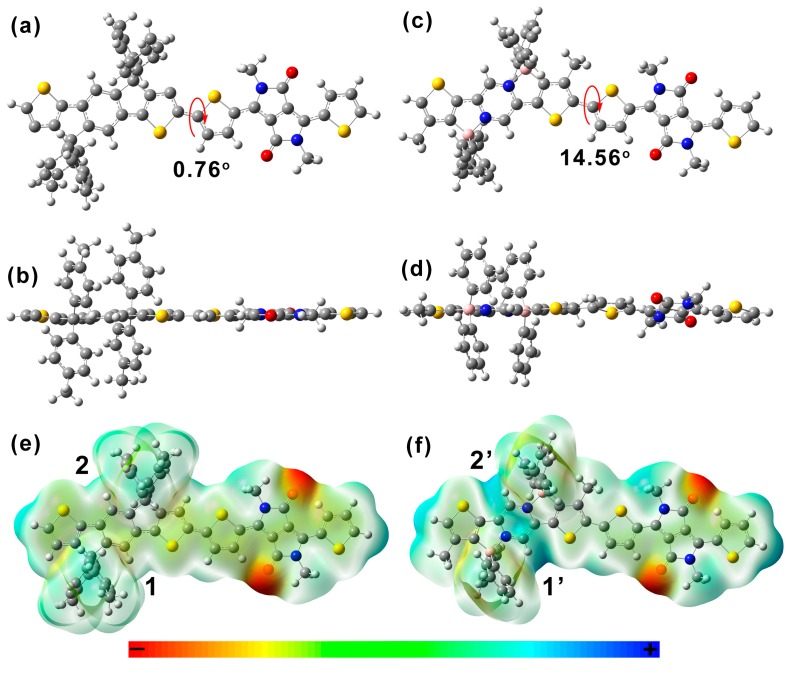
Optimized conformation and electrostatic potential surface (EPS) maps of IDT-DPP (**a,b**, and **e**) and BNIDT-DPP (**c,d**, and **f**) repeating units.

Gaussian simulations (Gaussian Inc., Wallingford, USA) were performed on one repeating unit at the B3LYP/6-31G (d, p) levels. The optimized conformation IDT-DPP repeating unit shows good backbone co-planarity (Figure 3a,b) with a small dihedral of 0.76° between IDT and DPP units. However, for BNIDT-DPP, a more distorted backbone is observed with a dihedral of 14.56° between BNIDT and DPP units (Figure 3c,d), resulting from the steric effect of alkyl chains anchoring on the β-position of the BNIDT unit. As for from IDT-DPP to BNIDT-DPP, the structural differences, including the backbones and side chains, we first studied the influence of β-substituted methyl groups on the electrostatic potential. We calculated the electrostatic potential surface (EPS) maps of two similar units, i.e., IDT and IDT-Me (Figure S2 in the supplementary material), revealing that the general electrostatic potential of the β-position is not affected by the methyl groups. The EPS maps of the IDT-DPP backbone display a general negative charge (–), indicating its electron-rich nature. In sharp contrast, after the B←N bonds were embedded, the BNIDT-DPP backbone becomes positively charged (+), demonstrating the electron-deficient nature. Especially from 1 and 2 to 1′ and 2′ positions, the electrostatic potential increases from a negative charge (yellow color) to an extremely positive charge (blue color), which can be readily ascribed to the introduction of B←N bonds. These diversities suggest that replacing the C–C bonds with B←N bonds could increase the electrostatic potential from a negative to a positive feature and enhance the electron affinity of the polymer backbone, supporting the decreased FMO energy levels and bandgaps.

**Figure 4:**
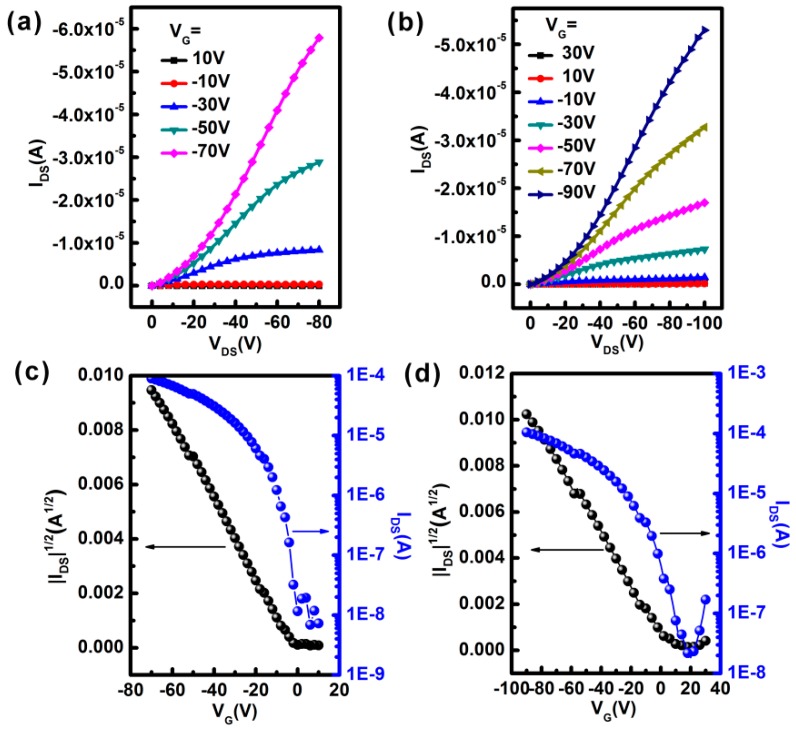
Output and transfer curves for IDT-DPP (**a**,**c**) and BNIDT-DPP (**b**,**d**).

Organic thin film transistors (OTFTs) adopting a top gate/bottom contact (TGBC) configuration were fabricated with IDT-DPP and BNIDT-DPP to study their semiconductor properties. As shown in Figure 4a,b, both the two polymers exhibit p-type semiconductor characteristics with well-defined output curves. The hole mobilities (*μ*_h_) calculated from the transfer curves (Figure 4c,d) are 0.059 and 0.035 cm^2^/V·s, respectively for IDT-DPP and BNIDT-DPP. The on/off ratios (*I*_on/off_) are 4.80 × 10^3^ and 1.30 × 10^4^, respectively for IDT-DPP and BNIDT-DPP. In general, the *μ*_h_ and *I*_on/off_ values of BNIDT-DPP are inferior to these of IDT-DPP. This can be ascribed to two points. On the one hand, the significantly higher molecular weight of IDT-DPP in contrast to that of BNIDT-DPP (92.1 versus 27.8 kDa) is favorable for the enhanced hole mobilities and *I*_on/off_ values. On the other hand, the worse backbone of BNIDT-DPP caused by the alkyl groups on the β-position of BNIDT, as evidenced by the Gaussian calculations, can be bad for the molecular packing order in solid films and deteriorate the device performance, as will be confirmed by the XRD data. It’s worth noting that although the BNIDT-DPP has relatively strong electron affinity, no n-type transport characteristic was observed based on the OTFT tests. This can be attributed to the unfavorable LUMO level and limited test conditions. It’s been well established that the deep LUMOs, as low as ca. −4.0 eV, are required for n-type OTFT properties, which is advantageous for electron injection, stable transport, and resisting charge carrier trapping under air conditions [46,47,48]. Although the LUMO of BNIDT-DPP (−3.74 eV) is relatively deeper than that of IDT-DPP (−3.54 eV), it′s not sufficient for the n-type charge transport. In addition, for most of the n-type operations of OTFT devices, preparation and tests in inert atmosphere are usually required to avoid the effects of oxygen and moisture, which cannot be fulfilled in our tests. 

**Figure 5:**
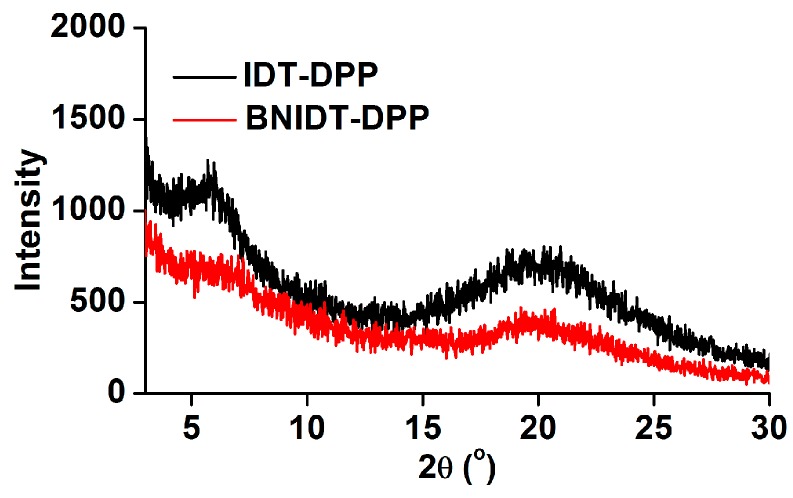
The XRD patterns of IDT-DPP and BNIDT-DPP in films.

The X-ray diffraction was tested to study the packing order of the polymers in films. As shown in Figure 5, IDT-DPP shows a lamellar packing (100) signal at 2θ = 5.83°, corresponding to the lamellar distance of 15.14 Å (Table 3). For BNIDT-DPP, a very weak 100 signal can be notice at 2θ = 6.91°, indicating a lamellar distance of 12.78 Å, which is shorter than the 100 distance of IDT-DPP, and can be attributed to the smaller side chain size of BNIDT-DPP. An apparent 010 diffraction signal at 2θ = 20.17° is observed for IDT-DPP, demonstrating a π–π distance of 4.4 Å. For BNIDT-DPP, the 010 signal is relatively weaker, with a π–π distance enlarged to 4.5 Å, presumably due to the worse backbone co-planarity of BNIDT-DPP, as revealed by the Gaussian simulations. Generally, the packing order in both the 100 and 010 directions of BNIDT-DPP is inferior to that of IDT-DPP, which can be ascribed to the lower molecular weight and the worse backbone co-planarity of BNIDT-DPP, and is also consistent with its lower OTFT performance.

**Figure 6:**
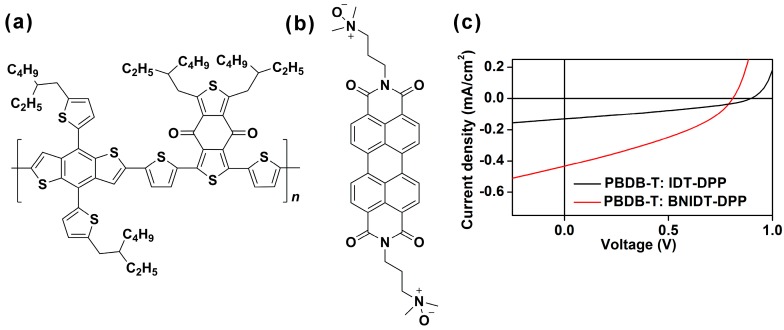
Molecular structures of PBDB-T (**a**) and PDINO (**b**) and *J*-*V* curves of all-PSCs based on IDT-DPP and BNIDT-DPP (**c**).

Considering the strong electron affinity of BNIDT-DPP, we prepared all-polymer solar cells (all-PSCs) by selecting BNIDT-DPP as the electron acceptor and PBDB-T (Figure 6a) as the electron donor. PDINO was selected as the electron-transport layer (Figure 6b) [49]. Preliminary device preparation by using CB as the spin-coating solvent affords an open-circuit voltage (*V*_OC_) of 0.81 V, a short-circuit current density (*J*_SC_) of 0.43 mA/cm^2^, a fill factor (FF) of 35.42%, and a power conversion efficiency (PCE) of 0.12% (Figure 6c). For comparison, IDT-DPP was also used as the acceptor for all-PSCs. In contrast to the BNIDT-DPP, the IDT-DPP-based device shows enhanced *V*_OC_ of 0.89 V, which can be readily ascribed to the relatively high-lying LUMO of IDT-DPP. Otherwise, the *J*_SC_ (0.13 mA/cm^2^) and the FF (33.59%) values of the IDT-DPP-based devices are apparently inferior to those of the BNIDT-DPP-based device. This can be attributed to the unmatchable energy level alignment between PBDB-T and IDT-DPP, with the HOMO of the acceptor higher than the donor by ca. 0.2 eV (Figure 2d) [44]. Although they have a broad and strong UV-vis-NIR light absorption, the small molecule acceptor based on the IDT and DPP units revealed in the literature also exhibited low efficiencies (PCE < 1%) by using P3HT as the donor polymer [50]. In general, the poor performance of IDT and DPP-based acceptors are mainly due to their unfavorable energy levels and poor electron mobilities. For BNIDT-DPP, although it possesses strong electron affinity and light-harvesting ability, the electron mobilities are also too low to be tested by OFETs, which is a hinder for the acceptor material. Accordingly, we anticipate that BNIDT-DPP will be useful as the third component for the ternary solar cells to enhance the light absorption and act as the energy bridge. This tentative work is undertaken in our group. 

## 3. Conclusions

We synthesized a B←N embedded polymer BNIDT-DPP along with a C–C counterpart IDT-DPP for comparison. The UV-vis-NIR absorption tests demonstrate the extended absorption spectra and narrowed optical bandgap (1.27 versus 1.48 eV) of BNIDT-DPP in contrast to those of IDT-DPP. Electrochemical tests indicate the depressed HOMO/LUMO energy levels for BNIDT-DPP (−5.22/−3.74 eV) in comparison to the IDT-DPP (−5.08/−3.54 eV). Theoretical simulations disclose that by replacing the C–C bonds with B←N bonds in the backbone, the electrostatic potential transforms from electron-negative (–) to electron-positive (+) feature, supporting the enhanced electron affinity of the B←N embedded polymer BNIDT-DPP, which is consistent with its decreased energy levels and bandgap. OTFT devices were prepared and tested by using TGBC configuration. Both IDT-DPP and BNIDT-DPP exhibit well-defined p-type semiconductor characteristics. Hole mobilities of IDT-DPP and BNIDT-DPP were tested to be 0.059 and 0.035 cm^2^/V s, respectively. Preliminary preparation of all-polymer solar cells by using BNIDT-DPP as the electron acceptor and PBDB-T as the electron donor reveals a *V*_OC_ of 0.81 V, a *J*_SC_ of 0.43 mA/cm^2^, a FF of 35.42%, and a PCE of 0.12%. This work develops a new class of B←N embedded polymer with strong electron affinity and extended absorption, which is potentially useful for electronic device application.

## Figures and Tables

**Table 1:** Molecular weight and thermal parameters of IDT-DPP and BNIDT-DPP.

Compounds	*M*_n_(kDa)^*a*^	*M*_w_(kDa)^*b*^	*M*_w_/*M*_n_^*c*^	*M*_r_(kDa)^*d*^	DP^*e*^	*T*_d_(°C)^*f*^
**IDT-DPP**	92.1	192.6	2.09	1.43	64	294
**BNIDT-DPP**	27.8	66.9	2.41	1.28	22	253

Notes: *^a^* Number-average molecular weight; *^b^* Weight-average molecular weight; *^c^* Polydispersity index; *^d^* Molecular weight of one repeating unit; *^e^* Degree of polymerization; *^f^* 5% Weight-loss temperature.

**Table 2:** Optical and energy levels parameters of IDT-DPP and BNIDT-DPP.

Compounds	^*a*^*λ*_max_^*s*^ (nm)	^*b*^*λ*_max_^*f*^ (nm)	^*c*^*λ*_edge_^*s*^ (nm)	^*d*^*λ*_edge_^*f*^ (nm)	^*e*^FWHM^*s*^ (nm)	^*f*^FWHM^*f*^ (nm)	^*g*^*E*_g_^opt^ (eV)	^*h*^HOMO (eV)	^*h*^LUMO (eV)	^*i*^*E*_g_^CV^ (eV)
IDT-DPP	720	715	825	836	110	158	1.48	−5.08	−3.54	1.54
BNIDT-DPP	784	760	929	978	277	307	1.27	−5.22	−3.74	1.48

Notes: *^a^* Absorption maxima in solution; *^b^* Absorption maxima in film; *^c^* Absorption edge in solution; *^d^* Absorption edge in film; *^e^* Full width at half maximum in solution; *^f^* Full width at half maximum in film; *^g^* Optical bandgap estimated from 1240/*λ*_edge_*^f^*; *^h^ E*_HOMO/LUMO_ = −(4.8 − *E*_(Fc0/+)_ + *E*_ox/red_^onset^), *E*_(Fc0/+)_ is 0.43 V; *^i^* Electrochemical bandgap. HOMO: highest occupied molecular orbital, LUMO: lowest unoccupied molecular orbital.

**Table 3:** OTFT and XRD parameters of IDT-DPP and BNIDT-DPP.

Compounds	*μ*_h_(cm^2^ V^–1^ s^–1^)	*I*_on_/*I*_off_	*V*_th_(V)	Lamellar Spacing(Å)	π–π Spacing(Å)
**IDT-DPP**	0.059	1.3 × 10^4^	−3	15.1	4.4
**BNIDT-DPP**	0.035	4.8 × 10^3^	3	12.8	4.5

## Supplementary Materials

The following are available online at https://www.mdpi.com/2073-4360/11/10/1630/s1. Device preparation and tests, material synthetic procedures and identification, Figure S1-S2, ^1^H NMR and ^13^C NMR spectra.
