# Supplementary material for: Synthesis of Conjugated Polymers Containing B←N Bonds with Strong Electron Affinity and Extended Absorption
Source: Polymers (Basel). 2019 Oct 9;11(10):1630. doi: 10.3390/polym11101630 (PMC6835370; doi:10.3390/polym11101630)
Supplement: Supplementary file 1 [file polymers-11-01630-s001.pdf]

Supporting Information for

Synthesis of Conjugated Polymers Containing B←N Bonds with Strong Electron Affinity and Extended Absorption

Bo Pang [1], *Zhonghai Tang* [2], *Yongchun Li* [1,2], *Huifeng Meng* [1,2], *Ying Xiang* [1], *Yuqing Li* [1,2] *and Jianhua Huang* [1],*

[1] College of Materials Science and Engineering, Huaqiao University, Xiamen, 361021, P. R. China.

[2] Beijing National Laboratory for Molecular Sciences, Institute of Chemistry, Chinese Academy of Sciences, Beijing 100190, P. R. China.

* Corresponding author: huangjianhua@hqu.edu.cn

Contents

1 General Methods for Measurements and Characterizations................................1

2 Gaussian Calculations 1

3 Fabrication and Tests of Photovoltaic devices...2

4 Experimental section 3

5 Supporting Figures and Tables 5

6 References...9

1 General Methods for Measurements and Characterizations

The ^{1}H NMR and ^{13}C NMR spectra were measured on a Bruker AVANCE 500 MHz spectrometer using tetramethylsilane (TMS; δ = 0 ppm) as an internal standard. Thermogravimetric analysis (TGA) was recorded on DTG-60H at a heating rate of 10 ºC/min under nitrogen flow. UV-vis-NIR absorption was recorded on a Shimadzu UV-2100 spectrophotometer (200–1000 nm). The electrochemical measurements were carried out in a deoxygenated solution of tetra-n-butylammonium hexafluorophosphate (Bu_4NBF_6, 0.1 M) in acetonitrile with a computer-controlled Zennium electrochemical workstation. A glassy carbon electrode, a Pt wire, and saturated calomel electrode (SCE) electrode were used as the working, counter, and reference electrodes, respectively. Ferrocene/ferrocenium (Fc/Fc^+) redox pair was used as the internal standard. The scanning rate was 100 mV/s.

2 Gaussian Calculations

Density functional theory (DFT) calculations were performed using the Gaussian 09 program with the B3LYP exchange-correlation functional.[3-5] All-electron triple-ξ valence basis sets with polarization functions (6-31G (d, p)) are used for all atoms. Geometry optimizations were performed with full relaxation of all atoms. All alkyl chains were reduced to methyl groups for avoiding computational load. For each molecule, various conformations with different dihedral angles were optimized, and the data for the one with the lowest energy are reported. HOMO and LUMO orbitals were deduced from cub files on Gaussian view. The electrostatic potential surface (EPS) maps were exported from fchk files on Gaussian view linked to Gaussian 09 program.

3 Experimental section

OTFTs with top gate/bottom contact (TGBC) geometry were successfully fabricated on heavily doped silicon wafers covered with thermally grown 300 nm thick silicon dioxide (SiO_2) layers. Gold source/drain electrodes were prepared by photolithography. Then the electrodes were dipped into acetone for 3 h and blow-dried by N_2 followed by dipping into the H_2SO_4/H_2O_2 (2:1) mix solvent. Next, deionized water, ethanol, and acetone were applied to wash the SiO_2 substrates. Then, the substrates were dried in atmosphere at 50 ºC. Octadecyltrichlorosilane (OTS) self-assembled layer was formed by using OTS solution in vacuum at 120 ºC. The treated substrates were rinsed with hexane, ethanol and chloroform. The thin film of the organic semiconductors was deposited onto the octadecyltrichlorosilane (OTS)-modified SiO_2 substrates by spin-coating a IDT-DPP or BNIDT-DPP solution (8 mg/ml), in which the semiconductors dissolved in o-dichlorobenzene. Thermal

annealing was performed in a nitrogen-filled glove box for 5 minutes at 200 ºC. Then, PMMA (M_w = 996 KDa) solution in anhydrous n-butyl acetate (60 mg/mL) was spin-coated onto the polymer followed by drying at 80 ºC for 30 min. finally, the Al (ca. 80 nm) gate was deposited under vacuum. The field-effect characteristics of small molecule devices were determined with a Keithley 4200 SCS semiconductor parameter analyzer. The tests were conducted in air. The charge carrier mobility (μ) in saturation was calculated from equation:

$$I_{DS} = (W/2L)Ci\mu(V_{GS} - V_{TH})^2$$

where W/L is the channel width/length, C_i is the gate dielectric layer capacitance per unit area, and V_{GS} and V_{TH} are the gate voltage and threshold voltage, respectively. The channel length (L) and channel width (W) of the OTFT devices in our study are 5 and 1400 μm, respectively.

4 Fabrication and Tests of Photovoltaic devices

Photovoltaic devices with a conventional configuration of ITO/PEDOT: PSS/Active Layer/PDINO/Al was fabricated as follows: The ITO glasses were pre-cleaned with sequential ultrasonication in a soap-deionized water mixture deionized water, CMOS grade acetone and isopropanol in turn for 15 min, respectively. The washed substrates were further treated with oxygen plasma for 30 min to eliminate any remaining organic components. Then a thin layer (approximately 20 nm) of PEDOT: PSS (poly(3,4-ethylenedioxythiophene)-poly(styrenesulfonate)) was spin-coated at 6000 RPM for 30 s and baked in oven at 150 °C for 20 min. The photovoltaic layer (PBDB-T: acceptor) with 1: 1 ratio were spin-coated at 1500 rpm for 50 s and then thermally annealed at 100 °C for 10 min. Atop of active layer a methanol solution of PDINO (optimized concentration 1 mg/ml) was spin-coated with rotation speed of 3000 rpm for 30 s. Finally Al (ca. 80 nm) was thermally deposited under the vacuum of 1×10^{-6} Torr. The active area of the device was 0.04 cm^2. The devices were characterized in nitrogen atmosphere under the illumination of simulated AM 1.5G (AAA grade, XES-70S1) light source. The illumination intensity in front of the cell sample was calibrated to be 100 mW/cm^2 with a reference silicon cell. The current density-voltage (*J*-*V*) measurement of the devices was recorded along the forward scan direction from -1.5 to 1.5 V using a computer-controlled Keithley 2400 Source Measure Unit.

5 Experimental section

All of the starting materials and solvents were purchased from Sigma Aldrich or TCI and were used without further purification, unless otherwise stated. All of the solvents were distilled from CaH_2 or Na and stored over activated 3Å molecular sieves. Compound (**1**) was prepared according to our previous work.[6]

Preparation of Ph_2Zn. In an oven-dried schlenk tube equipped with a stir bar, $ZnBr_2$ (1.19 g, 5.3 mmol) and LiCl (360 mg, 8.5 mmol) were added and the tube was heated under vacuum for 2 min with the help of a heat gun. After cooling down to room temperature, the THF (10 mL) was added in tube under the N_2 atmosphere and the mixture was stirred at room temperature for 5 min. Then PhMgBr (2.8 M in THF, 6 mL, 16.8 mmol) was added dropwise. The mixture was stirred at room temperature for 1 h. Then, the dense-grey solution was used directly in the next step without further treatment.

Synthesis of BNIDT. Under N_2 protection, compound **1** (500 mg, 0.9 mmol) was dissolved in dried CH_2Cl_2 (20 mL), triethylamine (Et_3N, 0.25 mL, 1.8 mmol) was added at 0 ºC. After stirring for 10 min, BBr_3 (0.52mL, 5.3mmol) was added dropwise. Then, the mixture was stirred at room temperature overnight under the N_2 atmosphere forming blue solution. Next, the prepared Ph_2Zn solution was added dropwise in the mixture at 0 ºC. The solution color was changed from blue to red-purple. Then, the mixture was stirred at room temperature overnight under the N_2 atmosphere. The solvent was removed by rotary evaporation. The mixture was extracted with ethyl acetate and water. Subsequently, the organic layer was dried over anhydrous $MgSO_4$, filtrated and evaporated using a rotary evaporator. The crude product was purified by silica gel column chromatography (petroleum ether/CH_2Cl_2 = 8:1). The product was recrystallized from n-hexane obtaining purple solid in 30% yield (206 mg, 0.27 mmol). ^{1}H NMR (500 MHz, CD_2Cl_2) δ 8.67 (s, 2H), 7.30 (s, 2H), 7.23 (s, 20H), 5.32 (s, 2H), 2.49 (t, *J* = 7.7 Hz, 4H), 1.31–1.22 (m, 4H), 1.19–1.04 (m, 12H), 0.80 (t, *J* = 7.2 Hz, 6H).

Synthesis of BNIDT-Br. BNIDT (100 mg, 0.13 mmol) and N-bromosuccinimide (NBS, 46 mg, 0.26 mmol) were dissolved in chloroform (12 mL) and degassed with N_2 for 10 min. The reaction system was sealed quickly. Then, the mixture was stirred at room temperature for 10 hours under N_2 atmosphere. The mixture was extracted with CH_2Cl_2 and H_2O. Then, the organic layer was dried over anhydrous $MgSO_4$, filtrated and evaporated using a rotary evaporator. The solid was purified by silica gel column chromatography (petroleum ether/dichloromethane = 10: 1). The product was obtained as purple-red solid with yield of 42%. H NMR (500 MHz, $CDCl_3$) δ 8.51 (s, 2H), 7.31–7.26 (m, 14H), 7.23 (dd, *J* = 6.5, 3.1 Hz, 7H), 2.49–2.43 (m, 4H), 1.13 – 0.87 (m, 19H), 0.78 (t, *J* = 7.3 Hz, 6H).

Synthesis of BNIDT-DPP. BNIDT-Br (260mg, 0.29mmol), DPP-Sn (250mg, 0.29mmol), and P(o-tolyl)$_3$ (6% mol) were dissolved in dry toluene (13mL) and degassed with N_2 for 8 min. Then Pd(PPh$_3$)$_4$ (3% mol) was added and degassed with N_2 for another 8 min. The reaction mixture was sealed quickly and heated to 110 ºC for 24 hours under N_2 atmosphere. After cooling to room temperature, the blocking agent 2-bromothiophene, 2-tributylstannylthiophene was sequentially added and reacted under N_2 atmosphere at 110 ºC for 1.5 h. The solution was added dropwise into methanol (200 mL). The precipitate was filtrated and collected. The collected solid was successively extracted with methanol, acetone, and hexane in a Soxhlet extractor to remove the monomers, oligomers, and other soluble organics. Finally, $CHCl_3$ was used to extract and the solution was condensed to 5mL, and then purified by silica gel column chromatography (Pure chloroform). The product was obtained as dark green solid with yield of 87%. ^{1}H NMR (500 MHz, $CDCl_3$) δ 9.04 (s, 2H), 8.59 (s, 2H), 7.38 (s, 2H), 7.31 (s, 19H), 4.06 (dd, *J* = 16.0, 9.2 Hz, 4H), 2.72 (s, 2H), 1.90 (s, 2H), 1.42–1.18 (m, 28H), 1.14–1.03 (m, 8H), 1.00–0.71 (m, 32H). BNIDTDPP-OC :^{1}H NMR (500 MHz, $CDCl_3$) δ 9.06 (s, 2H), 8.60 (s, 2H), 7.40 (s, 3H), 7.35–7.26 (m, 19H), 4.08 (s, 4H), 2.72 (s, 2H), 1.74 (s, 3H), 1.40 (d, *J* = 4.7 Hz, 6H), 1.34–1.18 (m, 32H), 1.16–0.75 (m, 39H). ^{13}C NMR (126 MHz, $CDCl_3$) δ 178.92, 161.65, 145.85, 144.55, 144.46, 144.39, 141.16, 140.99, 140.94, 139.49, 136.68, 134.07, 133.68, 131.27, 130.03, 129.72, 128.17, 127.99, 127.88, 127.63, 127.51, 127.20, 46.22, 39.25, 35.92, 31.95, 31.42, 30.22, 29.72, 29.59, 29.54, 29.34, 29.26, 29.13, 28.48, 27.24, 25.54, 24.70, 23.84, 23.59, 23.13, 10.27.

Synthesis of IDT-DPP. IDT-Sn (300 mg, 0.24 mmol), DPP-Br (164 mg, 0.24 mmol), and P(*o*-tolyl)$_3$ (6% mol) were dissolved in dry toluene (13mL) and degassed with N_2 for 8 min. Then Pd_2(dba)$_3$ (3% mol) was added and degassed with N_2 for another 8 min. The reaction mixture was sealed quickly and heated to 110 ºC for 24 hours under N_2 atmosphere. After cooling to room temperature, the blocking agent 2-bromothiophene, 2-tributylstannylthiophene was sequentially added and reacted under N_2 atmosphere at 110 ºC for 1.5 h. The solution was added dropwise into methanol (200 mL). The precipitate was filtrated and collected. The collected solid was successively extracted with methanol, acetone, and hexane in a Soxhlet extractor to remove the monomers, oligomers, and other soluble organics. Finally, $CHCl_3$ was used to extract and the solution was condensed to 5mL, and then purified by silica gel column chromatography (Pure chloroform) The product was obtained as dark green solid with yield of 85%.^{1}H NMR (500 MHz, $CDCl_3$) δ 8.88 (s, 2H), 7.46–7.32 (m, 8H), 7.20 (d, *J* = 18.3 Hz, 18H), 7.10 (d, *J* = 7.6 Hz, 15H), 2.57 (s, 19H), 1.90 (s, 5H), 1.46–1.10 (m, 107H), 0.97–0.78 (m, 60H). ^{13}C NMR (126 MHz, $CDCl_3$) δ 161.88, 160.83, 160.05, 157.20, 141.81, 141.28, 139.11, 138.98, 136.34, 135.58, 134.94, 133.99, 128.50, 127.86, 108.66, 63.07, 39.22, 36.77, 35.59, 31.94, 31.73, 31.36, 30.35, 29.72, 29.17, 28.56, 23.69, 23.10, 22.61, 14.11.

6 Supporting Figures

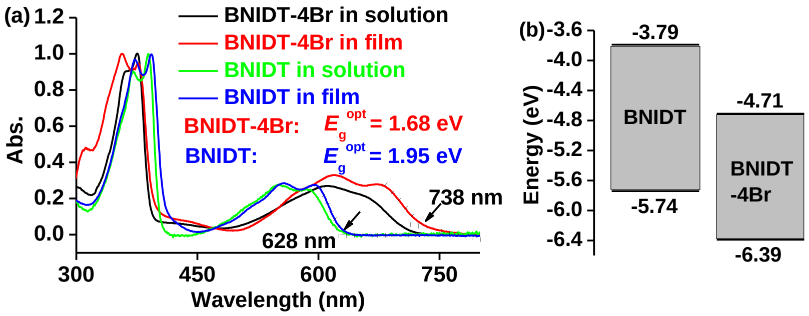

Figure S1. Absorption spectra of BNIDT-4Br and BNIDT in solutions and films (a) and the FMO energy levels of the two molecules. These data are from our previous work, Tetrahedron 2018, 74, 4308-4314 and Adv. Mater. in publishing.

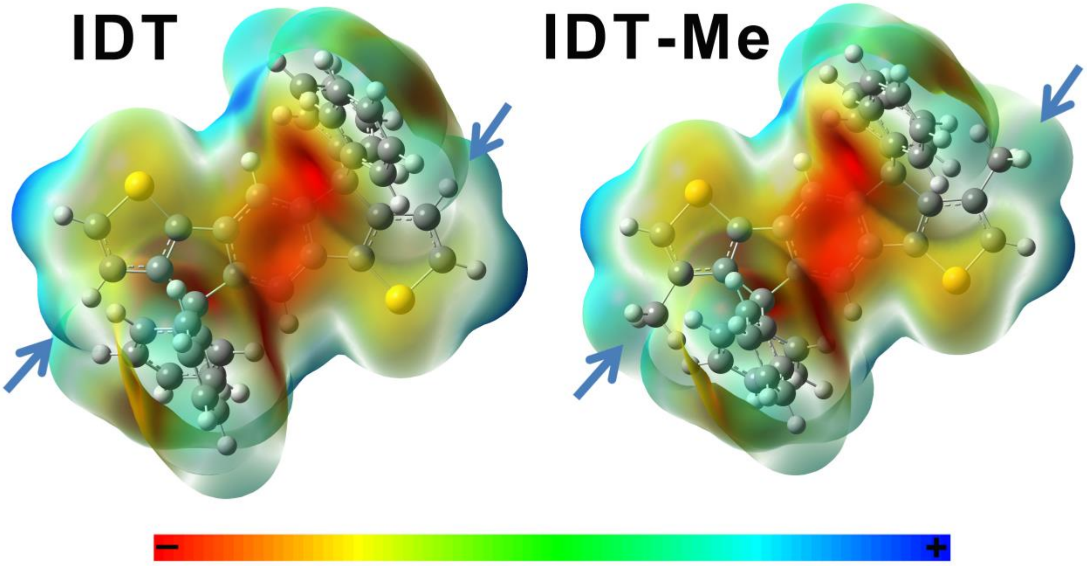

Figure S2. The EPS maps of IDT and IDT-Me. Arrow-marked positions represent the ß-positions substituted by methyl (Me) groups.

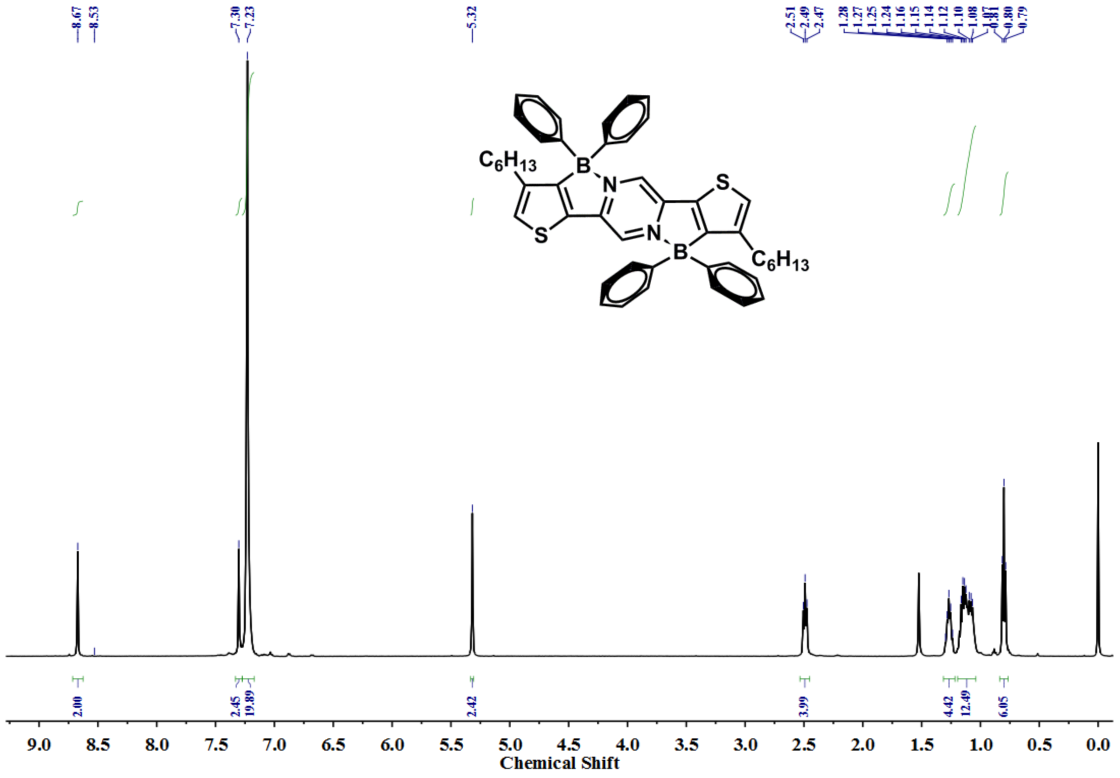

Figure S3. ^{1}HNMR spectra of BNIDT

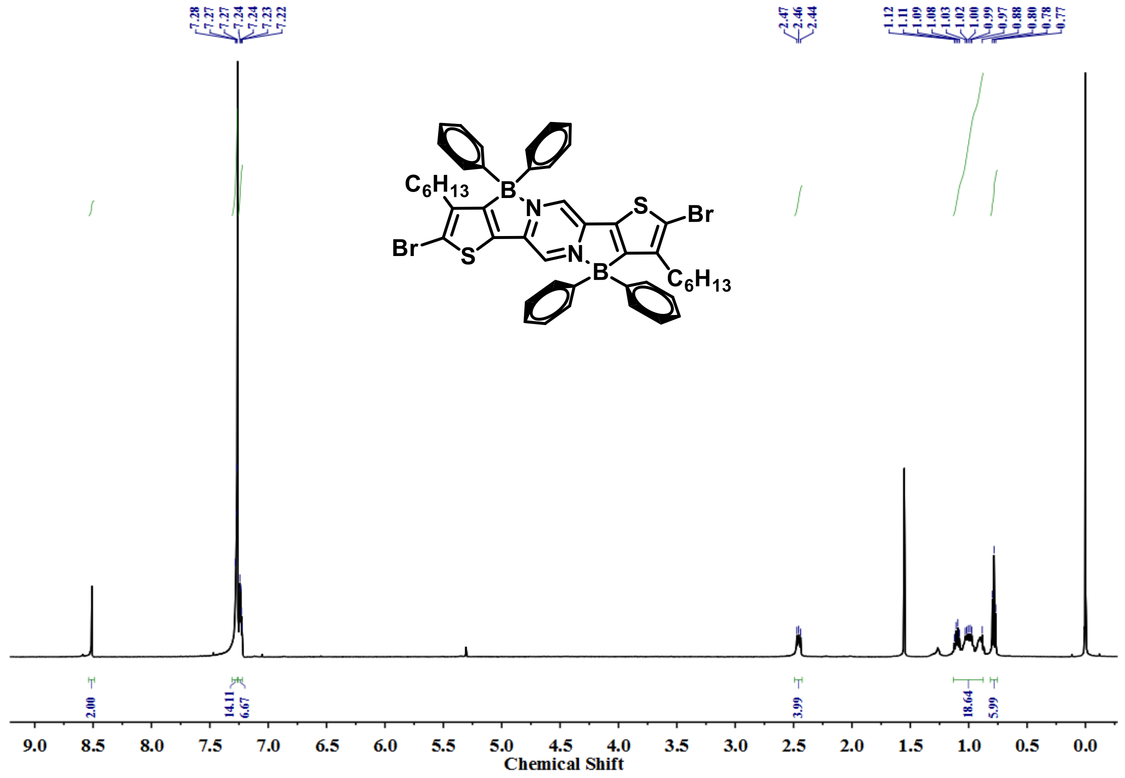

Figure S4. ^{1}HNMR spectra of BNIDT-Br

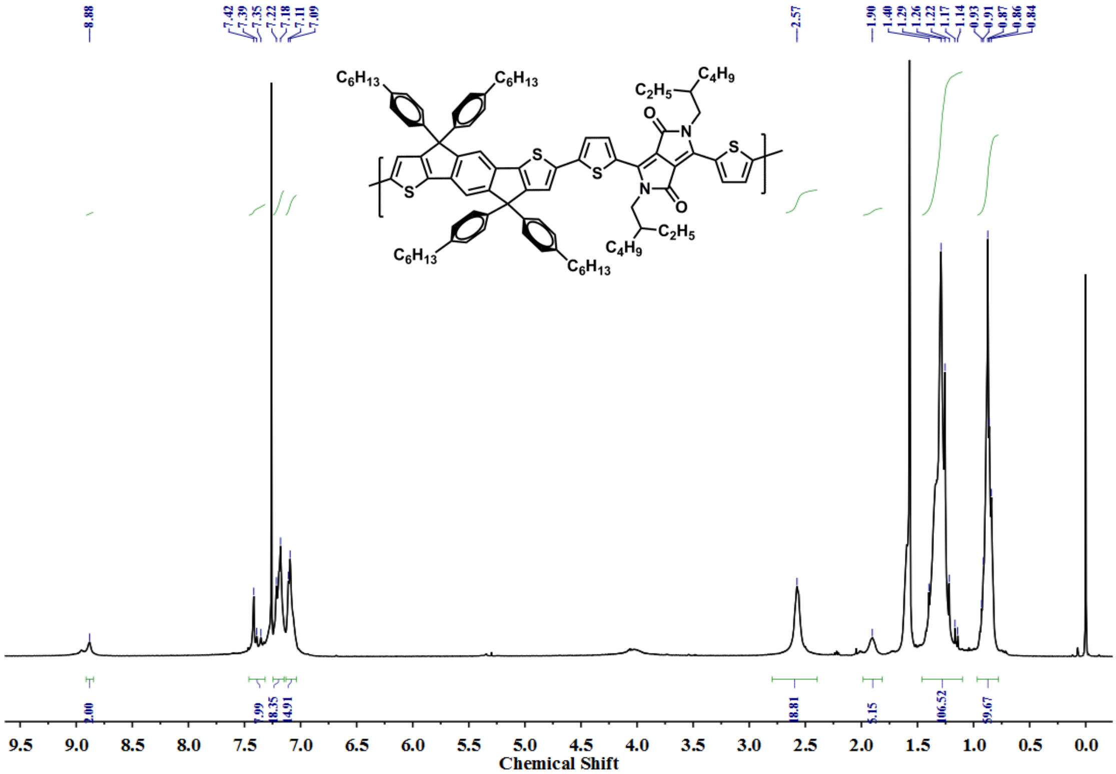

Figure S5. ¹HNMR spectra of IDT-DPP

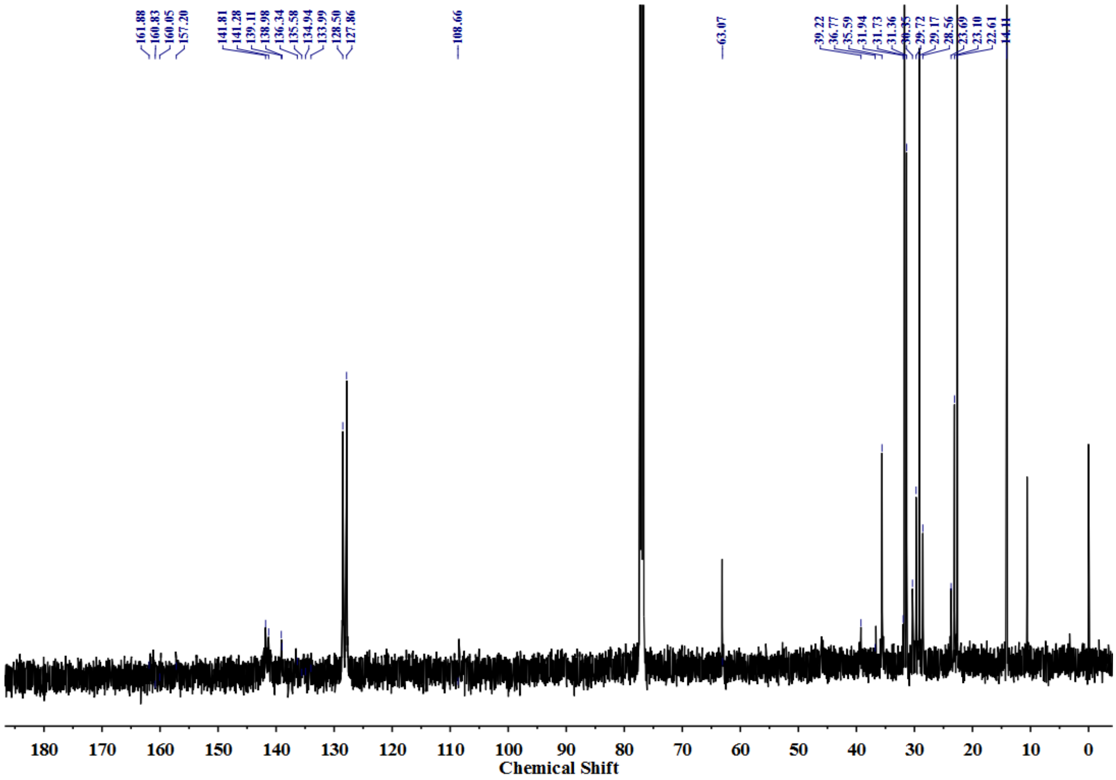

Figure S6. ¹³C NMR spectra of IDT-DPP in $CDCl_3$

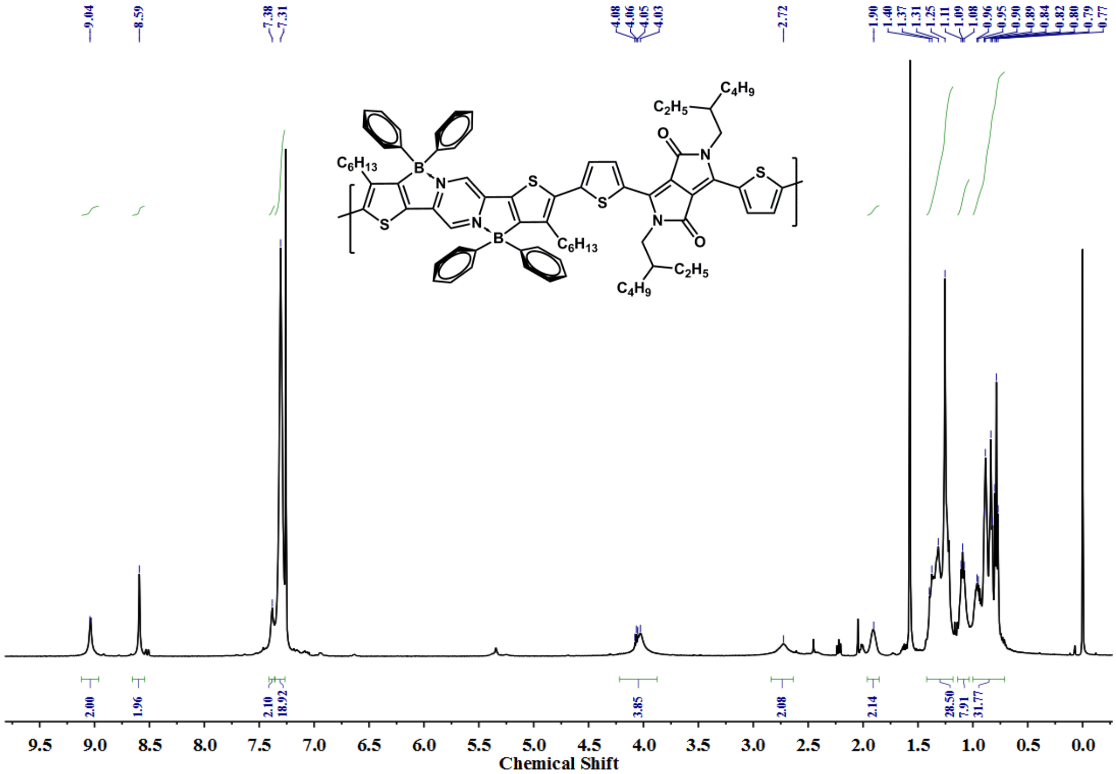

Figure S7. ^{1}HNMR spectra of BNIDTDPP-EH

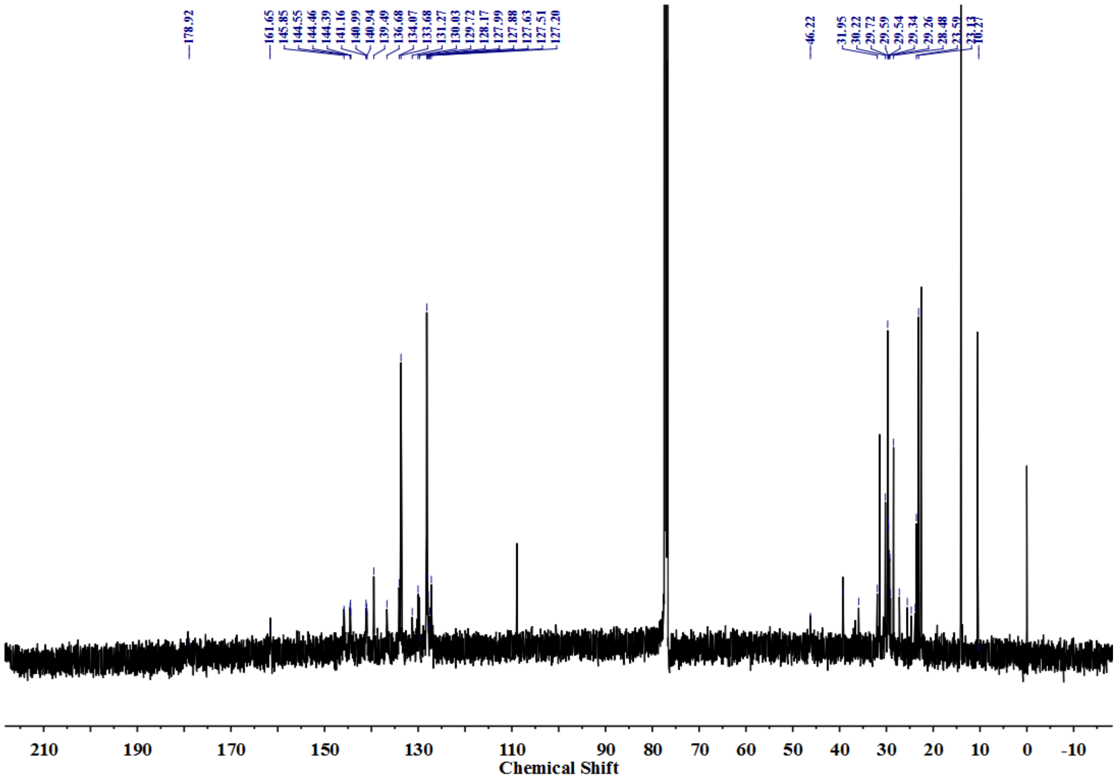

Figure S8. ^{13}C NMR spectra of BNIDTDPP-EH in $CDCl_3$

7 References

1. Olmsted, J., Calorimetric determinations of absolute fluorescence quantum yields. *J. Phys. Chem.* **1979,** *83*, 2581-2584.
2. Pan, Z. H.; Luo, G. G.; Zhou, J. W.; Xia, J. X.; Fang, K.; Wu, R. B., A simple BODIPY-aniline-based fluorescent chemosensor as multiple logic operations for the detection of pH and CO2 gas. *Dalton. Trans.* **2014,** *43,* 8499-507.
3. M. J. Frisch, G. W. T., H. B. Schlegel, G. E. Scuseria, M. A. Robb, J. R. Cheeseman, J. A. Montgomery Jr., T. Vreven, K. N. Kudin, J. C. Burant, J. M. Millam, S. S. Iyengar, J. Tomasi, V. Barone, B. Mennucci, M. Cossi, G. Scalmani, N. Rega, G. A. Petersson, H. Nakatsuji, M. Hada, M. Ehara, K. Toyota, R. Fukuda, J. Hasegawa, M. Ishida, T. Nakajima, Y. Honda, O. Kitao, H. Nakai, M. Klene, X. Li, J. E. Knox, H. P. Hratchian, J. B. Cross, V. Bakken, C. Adamo, J. Jaramillo, R. Gomperts, R. E. Stratmann, O. Yazyev, A. J. Austin, R. Cammi, C. Pomelli, J. W. Ochterski, P. Y. Ayala, K. Morokuma, G. A. Voth, P. Salvador, J. J. Dannenberg, V. G. Zakrzewski, S. Dapprich, A. D. Daniels, M. C. Strain, O. Farkas, D. K. Malick, A. D. Rabuck, K. Raghavachari, J. B. Foresman, J. V. Ortiz, Q. Cui, A. G. Baboul, S. Clifford, J. Cioslowski, B. B. Stefanov, G. Liu, A. Liashenko, P. Piskorz, I. Komaromi, R. L. Martin, D. J. Fox, T. Keith, M. A. Al-Laham, C. Y. Peng, A. Nanayakkara, M. Challacombe, P. M. W. Gill, B. Johnson, W. Chen, M. W. Wong, C. Gonzalez, and J. A. Pople, Gaussian 09 (Gaussian, Inc., Wallingford CT, 2009).
4. Becke, A. D., Density-functional exchange-energy approximation with correct asymptotic behavior. *Phys. Rev. A* **1988,** *38*, 3098-3100.
5. Lee, C.; Yang, W.; Parr, R. G., Development of the Colle-Salvetti correlation-energy formula into a functional of the electron density. *Phys. Rev. B* **1988,** *37*, 785-789.
6. Li, Y.; Meng, H.; Yan, D.; Li, Y.; Pang, B.; Zhang, K.; Luo, G.; Huang, J.; Zhan, C., Synthesis of B)N embedded indacenodithiophene chromophores and effects of bromine atoms on photophysical properties and energy levels. *Tetrahedron* **2018**, *74*, 4308-4314.
